# The why, what, who and when of B cell depletion in systemic lupus erythematosus: biological rationale, patient stratification and evolving therapeutic modalities

**DOI:** 10.1093/rheumatology/keag416

**Published:** 2026-08-14

**Authors:** Chris Wincup, Mariele Gatto, Marc Scherlinger, Georg Schett, Melanie Hagen

**Affiliations:** Department of Clinical and Academic Rheumatology, King’s College Hospital, London, UK; Centre for Rheumatic Diseases, King’s College London, London, UK; Academic Rheumatology Centre, Department of Clinical and Biological Sciences, University of Turin, Turin, Italy; Rheumatology Department, Centre de référence des maladies auto-immunes systémiques rares RESO, Hôpitaux Universitaires de Strasbourg, Strasbourg, France; Department of Medicine 3, Rheumatology and Immunology, Friedrich-Alexander-University Erlangen-Nürnberg (FAU) and Universitätsklinikum Erlangen, Erlangen, Germany; Deutsches Zentrum Immuntherapie (DZI), Friedrich-Alexander-University Erlangen-Nürnberg (FAU) and Universitätsklinikum Erlangen, Erlangen, Germany; Department of Medicine 3, Rheumatology and Immunology, Friedrich-Alexander-University Erlangen-Nürnberg (FAU) and Universitätsklinikum Erlangen, Erlangen, Germany; Deutsches Zentrum Immuntherapie (DZI), Friedrich-Alexander-University Erlangen-Nürnberg (FAU) and Universitätsklinikum Erlangen, Erlangen, Germany

**Keywords:** systemic lupus erythematosus, B cells, autoimmunity, B cell depletion, biomarkers, precision medicine, CAR-T, plasma cells, T cell engagers, biologics

## Abstract

Systemic lupus erythematosus (SLE) is driven by interconnected immune circuits in which B cells function as antigen-presenting cells, cytokine producers and precursors of autoantibody-secreting plasma cells. Therapeutic targeting of B cells aims to disrupt these self-reinforcing circuits rather than simply reduce autoantibody titres alone. Clinical experience with anti-CD20 monoclonal antibodies has validated B cells as therapeutic targets, while exposing key biological constraints, including incomplete tissue depletion, persistence of long-lived plasma cells, and BAFF-driven B cell repopulation. Recent advances including next-generation anti-CD20 monoclonal antibodies, bispecific T cell engagers and chimeric antigen receptor (CAR) T cell therapies have shown promise but questions remain relating to which patients should be treated with what modality of therapy and when this should be timed within the disease course. Herein, we evaluate the current landscape of B cell targeting therapies with particular focus on patient selection, timing of therapy and type of treatment.

Rheumatology key messages

- B cells sustain lupus through autoantibody production, antigen presentation, cytokine production and innate immune sensing.
- Efficacy of B cell depletion efficacy depends on the depth of B cell killing and the dominant pathogenic compartments.
- Future biomarker-driven stratification, including tissue profiling, will define precision B cell-depleting therapy in lupus.

## Introduction

Systemic lupus erythematosus (SLE) is a severe autoimmune disease characterized by loss of immune tolerance to nuclear antigens resulting in sustained activation of innate and adaptive immune pathways [1]. Although multiple immune compartments contribute to the pathogenesis of SLE, B cells occupy a central role. In SLE, B cells have been demonstrated to be capable of integrating antigen-specific recognition with innate immune sensing [2, 3], presenting antigen to T cells [4], producing pro-inflammatory cytokines [5] and differentiating into autoantibody-secreting plasma cells [6]. These functions place B cells at the centre of self-reinforcing immune circuits that sustain chronic inflammation and tissue damage in SLE.

Therapeutic targeting of B cells has therefore long been an attractive strategy in SLE. Clinical experience with B cell-depleting monoclonal antibodies has validated this approach, while simultaneously revealing important biological constraints, including incomplete depletion of tissue-resident B cells [7], persistence of long-lived plasma cells [8] and BAFF-mediated repopulation that may re-select autoreactivity [9]. More recently, advances in antibody engineering, T cell redirecting therapies and cellular immunotherapies have expanded the therapeutic landscape, enabling deeper or qualitatively distinct perturbation of B cell driven immune circuits [10].

In this review, we explore five clinically relevant questions for B cell-directed therapy in SLE: (i) why B cell targeting is mechanistically compelling, (ii) which B cell compartments and pathways are most relevant, (iii) what therapeutic modality best matches the biology and clinical context, (iv) who is most likely to benefit, and (v) when in the disease course these strategies should be deployed.

## Why B cells matter in SLE: central drivers of pathogenic immune circuits

### Autoantibody production and immune complex-mediated injury

Autoantibody production is a defining feature of SLE and remains one of the most direct mechanisms by which B cells mediate tissue damage. Autoreactive antibodies directed against double-stranded DNA, nucleosomes, ribonucleoproteins and phospholipid-associated antigens form immune complexes that deposit in target organs, activate complement, and engage Fc-receptors on myeloid cells [3, 11, 12]. Immune complex deposition within organs, such as the joints and kidneys, drives complement activation, recruitment of inflammatory cells and progressive severe organ injury resulting in significant damage-related morbidity and mortality [13–15].

Human and animal studies demonstrate that autoantibodies are not merely biomarkers of disease but active mediators of pathology. Transfer of anti-dsDNA antibodies induce glomerular inflammation and gene expression changes in renal tissue, while depletion of antibody-secreting cells ameliorates disease in lupus-prone animal models [16]. Furthermore, additional autoantibodies may have a direct pathogenic effect such as the anti *N*-methyl-D-aspartate receptor (NMDA-R) or anti-aquaporin 4 antibodies in neurological SLE involvement [17, 18]. Importantly, long-lived plasma cells residing in bone marrow and inflamed tissues can continue to secrete pathogenic antibodies independently of ongoing antigen exposure, contributing to disease chronicity and therapeutic resistance [19, 20].

### Sensing of nuclear antigens via B cell receptor–Toll like receptor cooperation

A defining feature of autoreactive B cells in SLE is their ability to couple antigen-specific recognition with innate immune sensing [2]. Nuclear antigens bound by the B cell receptor (BCR) are internalized and delivered to endosomal compartments containing Toll-like receptors (TLR), enabling synergistic signalling [21, 22]. This BCR–TLR cooperation lowers their activation thresholds, promotes proliferation and differentiation, and drives class-switch recombination even in the absence of normal tolerance checkpoints [23]. Memory B cells and plasmablasts exhibit increased expression of TLR7 and TLR9, correlating with autoantibody titres and disease activity [24]. Genetic and functional studies further support the pathogenic role of this pathway with gain-of-function variants in TLR7 associated with severe lupus phenotypes, while interruption of TLR signalling in animal models attenuates disease [25]. In addition to coding variation in this pathway, X-chromosome gene dosage provides a compelling biological explanation for the striking female bias in SLE. TLR7 is X-linked, and while one X chromosome is normally silenced by X-chromosome inactivation (XCI), lymphocytes show unusually dynamic maintenance of XCI. In human B cells, epigenetic features of the inactive X can be attenuated or mislocalized, creating a permissive state for escape from XCI and increased expression of immunity genes [26]. Consistent with this, single-cell allelic analyses demonstrate that a substantial fraction of primary B cells can express TLR7 biallelically in females. Biallelic TLR7 expression correlates with higher TLR7 expression and enhanced TLR7-driven B cell functional responses, including increased enrichment during TLR7-mediated plasma cell differentiation and a greater propensity for IgG class-switching [27]. Together, this supports a model in which defective XCI (particularly within the B cell compartment) amplifies TLR7 dosage and signalling, synergizing with genetic gain-of-function and reinforcing a mechanistic basis for female-predominant susceptibility in TLR7-dependent lupus biology. These observations place autoreactive B cells at the interface of adaptive and innate immunity, acting as antigen-specific sensors of self-derived nucleic acids.

### Antigen presentation and amplification of autoreactive T cell responses

B cells also play a critical role as antigen-presenting cells in SLE with autoreactive B cells efficiently internalizing self-antigens via the BCR and presenting processed peptides to CD4⁺ T cells through MHC class II [28, 29]. This interaction is particularly important for sustaining T follicular helper (Tfh) and peripheral helper T (Tph) cell populations (with the latter being particularly relevant in inflamed non-lymphoid tissues in SLE), which in turn provide IL-21, CD40L and other signals that drive B cell differentiation and survival [30]. Evidence from murine lupus models demonstrates that B cells capable of antigen presentation, but not antibody secretion, can still sustain autoimmune disease, highlighting the importance of this function [31]. Expansion of Tfh-like populations has also been shown to correlate with plasmablast frequencies and disease activity, reinforcing the concept of bidirectional B–T cell collaboration as a central pathogenic axis [32].

### Cytokine production by pathogenic B cell subsets

Activated B cells in SLE contribute directly to the inflammatory milieu through cytokine production. B cell-derived IL-6 promotes Tfh differentiation and germinal centre activity, while TNF supports lymphoid architecture and follicular dendritic cell networks. Experimental models demonstrate that B cell-intrinsic IL-6 is required for spontaneous germinal centre formation and class-switched autoantibody production, positioning B cell cytokine output as a driver of adaptive immune dysregulation [5]. While regulatory B cells capable of producing IL-10 have been described, their function appears impaired in established SLE. Indeed, elevated IL-10 levels in patients may paradoxically support B cell survival and antibody production, underscoring the complexity of B cell-derived cytokine networks in lupus [33].

### Interplay with type I interferon

Type I interferon (IFN-I) represents a central amplifier of lupus pathogenesis, and its relationship with B cells is bidirectional [34]. IFN-I enhances B cell activation by upregulating TLR expression, lowering activation thresholds, and promoting differentiation toward plasmablast and plasma cell fates [35]. B cell-intrinsic IFN-α/β receptor (IFNAR) signalling is required for full development of autoreactive germinal centre and memory responses in lupus-prone mice [36]. Conversely, B cell-derived autoantibodies and immune complexes promote IFN-I production by plasmacytoid dendritic cells through Fc receptor and TLR engagement [37].

Clinically, this bidirectional axis frames an important stratification question that currently available evidence cannot yet resolve definitively. Patients with a dominant IFN-I signature may reasonably be considered for type I IFN receptor blockade treatment with anifrolumab, although importantly clinical trials were conducted with the therapy used as an ‘add on’ to standard of care, in which IFN signatures may be confounded by concurrent immunosuppressive treatment [38].

## Which B cells should be targeted? Lineage, compartment and therapeutic depth

**Figure 1:**
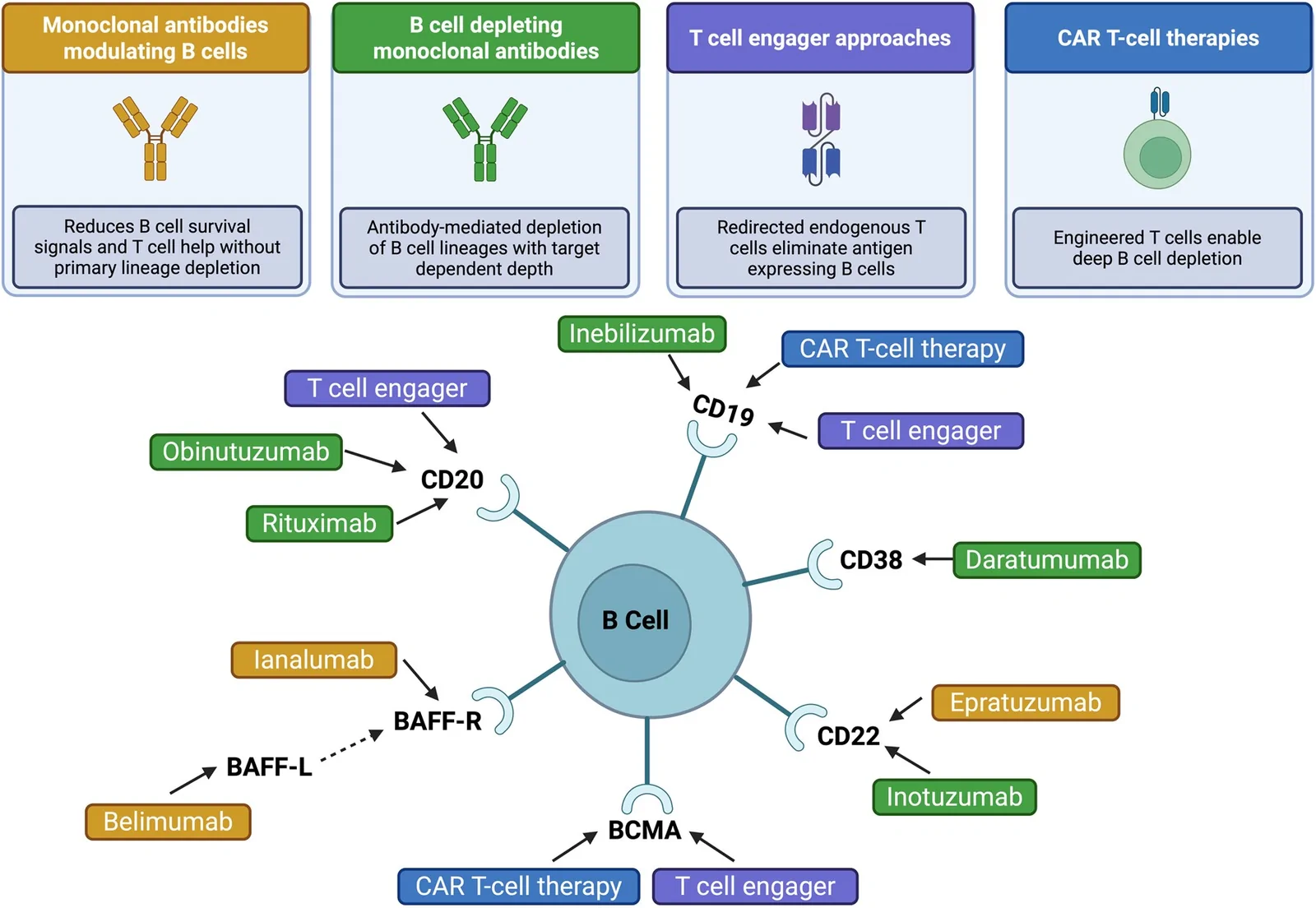
Current and potential B cell therapeutic targeting strategies in systemic lupus erythematosus. Abbreviations: BAFF: B cell activating factor; BAFF-L: B cell activating factor ligand; BAFF-R: B cell activating factor receptor; BCMA: B cell maturation antigen; CAR: chimeric antigen receptor. Created in BioRender. Wincup, C. (2026) https://BioRender.com/nno00v7

A complementary way to understand B cell-directed therapy is to map target antigen expression across the B cell lineage and compartments, then infer which pathogenic functions are interrupted and which effector populations are spared. These are summarized in Table 1 and Fig. 1. Importantly, B cell depletion is not a uniform intervention and can vary in its breath and depth. For instance, CD20 monoclonal antibodies predominantly eliminate circulating naïve and memory B cells, but spare plasmablasts and long-lived plasma cells due to their lack of CD20 expression, as well as tissue B-cells due to their lower susceptibility to therapeutic antibodies. In contrast, CD19 and BCMA-directed approaches extend depletion into plasmablast and plasma cell compartments, respectively. Furthermore, cell-based B cell depletion approaches have high potential to eliminate tissue B-cells and plasma cells. Therapeutic impact therefore depends on both B cell lineage targeting breadth and access to pathogenic tissue niches [39].

**Table 1:** B cell antigens involved in the immune mechanism of systemic lupus erythematosus.

Target antigen	Lineage expression	Key biological role in SLE	What targeting achieves	Major limitations/gaps	Therapeutic relevance
CD19	Pro-B cell until plasmablasts; absent on plasma cells	Positive regulator of BCR signalling	Broad B cell lineage depletion including plasmablasts	Spares long-lived plasma cells	Targeted by CAR-T cells, T cell engagers and anti-CD19 Mab inebilizumab
CD20	Pre-B cells until memory B cells; absent on plasmablasts/plasma cells	Positive regulator of BCR signalling; calcium channel activity	Broad B cell lineage depletion	Spares short-lived plasmablasts and long-lived plasma cells	Established biologic target using anti-CD20 Mab (rituximab, obinutuzumab)
CD21	Immature B cells until memory B cells; follicular dendritic cells; absent on plasmablasts/plasma cells	Co-receptor binding complement-tagged antigens	Broad B cell lineage depletion and targeting of follicular dendritic cells	Does not target SLE-associated activated memory B cells	No targeting drug available
CD22	Immature B cells until plasmablasts; absent on plasma cells	Inhibitory regulation of BCR signalling increasing threshold for B cell activation	Memory B cell and plasmablast depletion	Limited efficacy in highly amplified disease; modest depletion	Negative studies with anti-CD22 Mab epratuzumab in SLE, used as immunotoxin in lymphoma (inotuzumab)
CD38	Pro B-cells to immature B cells; plasmablasts and plasma cells, activated T cells and NK cells	Supports expanded antibody-secreting cells and early B cell development as co-receptor	Depletion of early B cells, plasmablasts, plasma cells and a subset of activated T cells	Hypogamma-globulinaemia; risk of infection	Anti-CD38 Mab daratumumab has shown efficacy in small case series in SLE; established target in multiple myeloma
CD40	Pro-B cells until plasmablasts; usually absent on plasma cells; expressed on other antigen-presenting cells (dendritic cells, macrophages)	Central co-stimulatory axis for T–B cells, germinal centre formation, class switching, affinity maturation, and memory/plasma-cell generation	Pathway interruption rather than depletion; reduces germinal-centre activity and downstream class-switching	No cell depletion approach; most likely immune balancing than resetting	Ligand (CD40L) targeted by Mab dapirolizumab as non-B cell-depleting strategy
BAFF-R	Immature B cells until plasmablasts; absent on plasma cells	Critical regulator of peripheral B cell survival and selection, especially at transitional and naïve stages; elevates survival thresholds	Broad B cell depletion including plasmablasts; blockade of B cell survival signals	No targeting of long-lived plasma cells; tissue-resident/GC-driven disease may be less BAFF-dependent	Ligand targeting (BAFF) by Mab belimumab is validated pathway; anti-BAFF-R Mab ianalumab under investigation
BCMA	Memory B cells, plasmablasts and plasma cells	Plasma-cell survival via APRIL/BAFF ligands; sustains autoantibody production	Direct elimination of antibody-secreting compartment	Hypogamma-globulinaemia; risk of infection	Targeted by CAR T cells and T cell engagers; established target in multiple myeloma

Abbreviations: APRIL: a proliferation-inducing ligand; ASC: antibody-secreting cell; BAFF: B cell activating factor; CAR: chimeric antigen receptor; EF: extrafollicular; GC: germinal centre; Mab: monoclonal antibody; NK: natural killer

### Germinal centre and extrafollicular B cell pathways

Pathogenic B cell responses in SLE have previously been demonstrated to arise through both germinal centre and extrafollicular (EF) pathways. Germinal centre reactions generate high-affinity, class-switched memory B cells and long-lived plasma cells, underpinning durable autoantibody production [40]. EF responses, driven by TLR engagement and IFN signalling, produce rapid short-lived plasmablast proliferation and are increasingly recognized in active and treatment-resistant disease [41]. These pathways are not mutually exclusive and may coexist within individual patients. Importantly, both are seeded by precursor populations that are susceptible to depletion, whereas their downstream products (particularly long-lived plasma cells) are relatively resistant to many therapies to date.

### Tissue-resident B cells and survival niches

Peripheral blood measurements substantially underestimate the complexity of B cell involvement in SLE. Pathogenic B cells reside in secondary lymphoid organs, inflamed tissues (such as the kidney and skin) and bone marrow plasma cell niches [42]. Tertiary lymphoid structures within target organs provide local sites for antigen presentation and differentiation, contributing to therapeutic resistance [43]. These observations have important implications for B cell-directed therapies. Apparent peripheral depletion of B lymphocytes in the blood does not necessarily equate to effective immunological control, and deeper or alternative strategies may be required to disrupt tissue-resident pathogenic compartments.

## What modality of B cell depletion: approaches to B cell-targeted therapy in systemic lupus erythematosus

Within the expanding modality landscape, an increasingly important distinction is whether depletion is directed primarily at the mature B cell compartment or extends into long-lived plasma cell pools. Anti-CD20 therapy, by design, leaves antibody-secreting cells largely untouched, whilst exerting effects through depletion of upstream immune-complex-amplifying B cell circuits and disruption B–T interaction. However, in a substantial subset of patients, persistent antibody production is maintained despite effective peripheral B cell depletion. This suggests long-lived plasma cells as the dominant residual pathogenic compartment. Proof-of-concept for plasma cell-directed therapy in SLE has been supported by limited studies on the anti-CD38 targeting monoclonal antibody, daratumumab, although there is an absence of clinical trial evidence [44]. BCMA-direct cellular therapies now offer a potentially more selective route to the plasma cell compartment, which provides a mechanistic case for compartment-led treatment selection (rather than sequential cycling of broader therapies), although greater biomarker development is required for such precision in clinical practice.

### Monoclonal antibody therapy

Anti-CD20 monoclonal antibodies (such as obinutuzumab and rituximab) deplete circulating and to some extent tissue B cells while sparing plasmablast and plasma cells, owing to loss of CD20 expression during terminal differentiation. Rituximab results in B cell depletion through a combination of antibody- and complement-dependent cytotoxicity [45]. Despite biological activity, rituximab failed to meet primary endpoints in both the EXPLORER [46] and LUNAR trials [47], possibly related to failure of monoclonal antibody to migrate into tissue. Post-hoc reanalysis of the EXPLORER trial addressed whether the apparent lack of response to rituximab in non-renal SLE reflected insensitivity of the original endpoints rather than lack of biological effect. Re-evaluation using contemporary composite outcomes again demonstrated no significant benefit over placebo, confirming that trial failure was not explained by end point choice. High background steroid and immunosuppressant use likely obscured incremental treatment effects, which is a recurring challenge in SLE trials. Nonetheless, exploratory subgroup analyses suggested potential benefit in select phenotypes (such as vasculitis or haematologic disease), reinforcing the view that rituximab efficacy in SLE may be context- and endotype-dependent rather than universally absent [48]. Notably, both vasculitic or haematological involvement in SLE involves compartments rich in cytotoxicity effectors, including Fc-receptor bearing cells and complement, which may favour effective antibody-mediated B cell killing. Rituximab has therefore never been approved for SLE but remained an option for refractory disease in EULAR guidelines [49]. In fact, extensive real-world experience, continues to demonstrate clinical benefit of rituximab in selected patients, particularly those with refractory organ-threatening disease, underscoring that lack of trial efficacy does not equate to lack of biological relevance [50–53].

Across studies of rituximab in SLE, the most consistent predictor of subsequent clinical response has been early and complete peripheral B cell depletion, rather than any single baseline serological or clinical marker [54]. Sensitive flow cytometric analyses have shown that patients who achieve near-complete depletion shortly after infusion are significantly more likely to respond than those with incomplete depletion [55, 56]. This observation reframes a proportion of apparent biologic non-responders as poor-depleters, with important therapeutic implications. Failure of rituximab therefore does not necessarily invalidate B cell targeting but may instead indicate insufficient pharmacodynamic effect (lack of B cell cytotoxicity), inappropriate B cell target selection (sparing CD20^low/−^ plasmablasts and plasma cells), or failure of the therapy to sufficiently penetrate involved tissue (resulting in only peripheral B cell depletion).

Equally important is the biology of B cell repopulation; for example, relapse following anti-CD20 therapy is often associated with rapid re-emergence of memory B cells and plasmablasts, rather than gradual reconstitution dominated by naïve cells [57, 58]. This pattern is biologically plausible with the increase of B cell activating factor (BAFF) concentrations after B cell depletion. Such a ‘BAFF-rich’ environment could stimulate residual memory B cells and trigger relapses based on re-selection of autoreactivity rather than loss of initial efficacy [9, 59].

Recognition of these limitations has driven development of next-generation anti-CD20 antibodies with enhanced effector function. Obinutuzumab is a type II, glycoengineered anti-CD20 monoclonal antibody designed to induce more potent antibody-dependent cellular cytotoxicity and phagocytosis, with reduced reliance on complement [60]. The effect of obinutuzumab in SLE is supported by positive findings of the phase III REGENCY trial, which demonstrated significantly higher complete renal response rates with obinutuzumab plus standard therapy compared with placebo (46.4% *vs* 33.1%) in lupus nephritis [61]. Importantly, these trials provide the strongest prospective evidence to date that deeper and more sustained B cell depletion can translate into improved clinical outcomes in LN leading to approval in combination with mycophenolate mofetil. Furthermore, this has subsequently been followed by the positive phase III ALLEGORY trial in extra-renal SLE in which 76.7% of participants achieved an SRI-4 response when compared with 53.5% who received placebo [62].

Post-hoc analysis from the NOBILITY trial of obinutuzumab in LN has demonstrated that complete renal response was accompanied by more profound and sustained peripheral B cell depletion, supporting depth of depletion as a key response-associated signal [63]. From a stratification perspective, anti-CD20 therapy is therefore best suited to patients in whom mature B cells remain central to disease propagation and in whom effective depletion can be achieved and confirmed.

Collectively, these data suggest that apparent non-response to anti-CD20 therapy often reflects insufficient depletion rather than absence of B cell-driven disease, reinforcing the need to develop novel strategies to induce deep B cell depletion.

Whilst monoclonal antibody therapy in SLE has predominantly centred on targeting CD20, inebilizumab is an instructive example of CD19 targeting B cell-depleting therapy that has been approved for aquaporin-4-IgG positive neuromyelitis optica spectrum disorder, where it reduces relapse risk [64]. Although CD19 targeting offers broader lineage coverage than CD20 (potentially extending into plasmablast-proximal compartments) there are currently limited data for use in SLE.

Another non-CD20-targeted B cell-depletion approach that is pursued in SLE is the BAFF-receptor (BAFF-R) directed antibody ianalumab. BAFF-R is expressed on a large range of B cell lineage cells including plasmablasts but not on plasma cells. This antibody combines functional blockade of BAFF-dependent survival signalling with antibody-dependent cellular cytotoxicity-mediated depletion of BAFF-R-expressing B cells. Through this mechanism of action, the drug exerts both modulatory and depleting effects on the B cell compartment. This dual mechanism is mechanistically attractive in SLE, as BAFF-R signalling is critical for the survival, selection and maturation of naïve and memory B cells and is upregulated in BAFF-rich inflammatory environments, particularly following B cell depletion, contributing to autoreactive repertoire reconstitution [59, 65]. In a phase II randomized trial in moderate–severe SLE, ianalumab significantly improved the primary composite end point of SRI-4 response with sustained corticosteroid tapering at week 28 compared with placebo, alongside consistent benefits across secondary outcomes including more stringent responder definitions, flare reduction and serological measures, with an acceptable safety profile [66]. These data support BAFF-R as a therapeutically actionable node in SLE and position ianalumab as a potential next-generation B cell-targeted therapy capable of integrating depletion with control of BAFF-driven B cell survival and repopulation dynamics.

### T cell engagers

T cell engagers (TCEs) are engineered molecules that bind B lineage cells (via CD19, CD20 or BCMA) and engage T cells, usually through binding CD3. By physically bridging T cells to the target cell, they enforce formation of an immune synapse, triggering T cell activation, proliferation, and cytotoxicity directed against B cells. The key conceptual distinction from conventional monoclonal antibodies is that TCEs do not rely on Fc effector functions (NK cells) but instead harness endogenous T cell cytotoxicity [67].

In SLE, and also other B cell driven autoimmune diseases, this approach offers several theoretical advantages: (i) CD20 × CD3 and CD19 × CD3 engagers could provide deeper and more uniform depletion of mature B cells across tissue compartments, while BCMA × CD3 engagers can directly target plasma cells; and (ii) T cell engagers are protein-based drugs, the production and administration of which follows the established procedures for monoclonal antibodies.

Early clinical exploration is underway in active SLE with a recent mechanistic case report describing rapid, drug-free remission of severe refractory SLE following short-course treatment with teclistamab, a BCMA × CD3 engager approved for multiple myeloma [68]. Further clinical experience with TCEs in autoimmune diseases has been promising [69–72]. Toxicity profiles of TCEs in autoimmune diseases seem to be acceptable with only low-grade cytokine-release syndrome, and no reports of neurotoxicity been found. Dosing and long-lasting clinical efficacy remain the most open questions to TCE treatments in SLE. First clinical trials are ongoing and will be essential to provide answers to these questions.

### Chimeric antigen receptor cell therapy

Treatment with chimeric antigen receptor (CAR) T cells differs from TCEs in a fundamental way. Instead of redirecting endogenous T cells with a therapeutic antibody, CAR T cell therapy engineers immune cells to express a new receptor that recognizes the target antigen on B cells. CAR-based therapies may be autologous (patient-derived T cells), allogeneic (derived from healthy donor cells) or *in vivo* (commonly done via the infusion of lipid nanoparticles carrying the payload encoding for the CAR). An overview of these approaches is summarized in Table 2.

**Table 2:** Comparative features of autologous, allogeneic, and *in vivo* CAR strategies.

CAR approach	Manufacturing	Access/scalability	Expansion/persistence	Potential toxicity	Challenges
Autologous CAR	Patient T cells collected → engineered *ex vivo* → reinfused	Moderate (individualized *ex vivo* manufacturing, slower turnaround)	High *in vivo* expansion of CAR T cells (low number of therapeutic cells necessary)	CRS and ICANS, lymphodepletion-related toxicity (haematotoxicity)	Complexity of procedure and necessity for lymphodepletion
Allogeneic CAR	Healthy donor cells → engineered → banked for use	High (ready availability; off-the-shelf; faster turnaround)	Low *in vivo* expansion of CAR T cells (high number of therapeutic cells necessary)	CRS and ICANS, lymphodepletion-related toxicity (hematotoxicity); graft rejection, graft-*vs*-host disease	Graft rejection due to immunogenicity
*In vivo* CAR	Delivery (via LNP) of CAR-encoding nucleic acid → T cells formed inside patient	Very high (no cell collection and no cell manufacturing)	Potentially low expansion of *in vivo* formed CAR T cells with dilution/loss of CAR with cell division (mRNA-based approach)	CRS and ICANS, hepatotoxicity	Sufficient numbers of CAR T cells and exposure time

Abbreviations: CAR: chimeric antigen receptor; CRS: cytokine release syndrome; GVHD: graft-*vs*-host disease; ICANS: Immune effector cell-associated neurotoxicity syndrome; LNP: lipid nanoparticle; QC: quality control.

**Figure 2:**
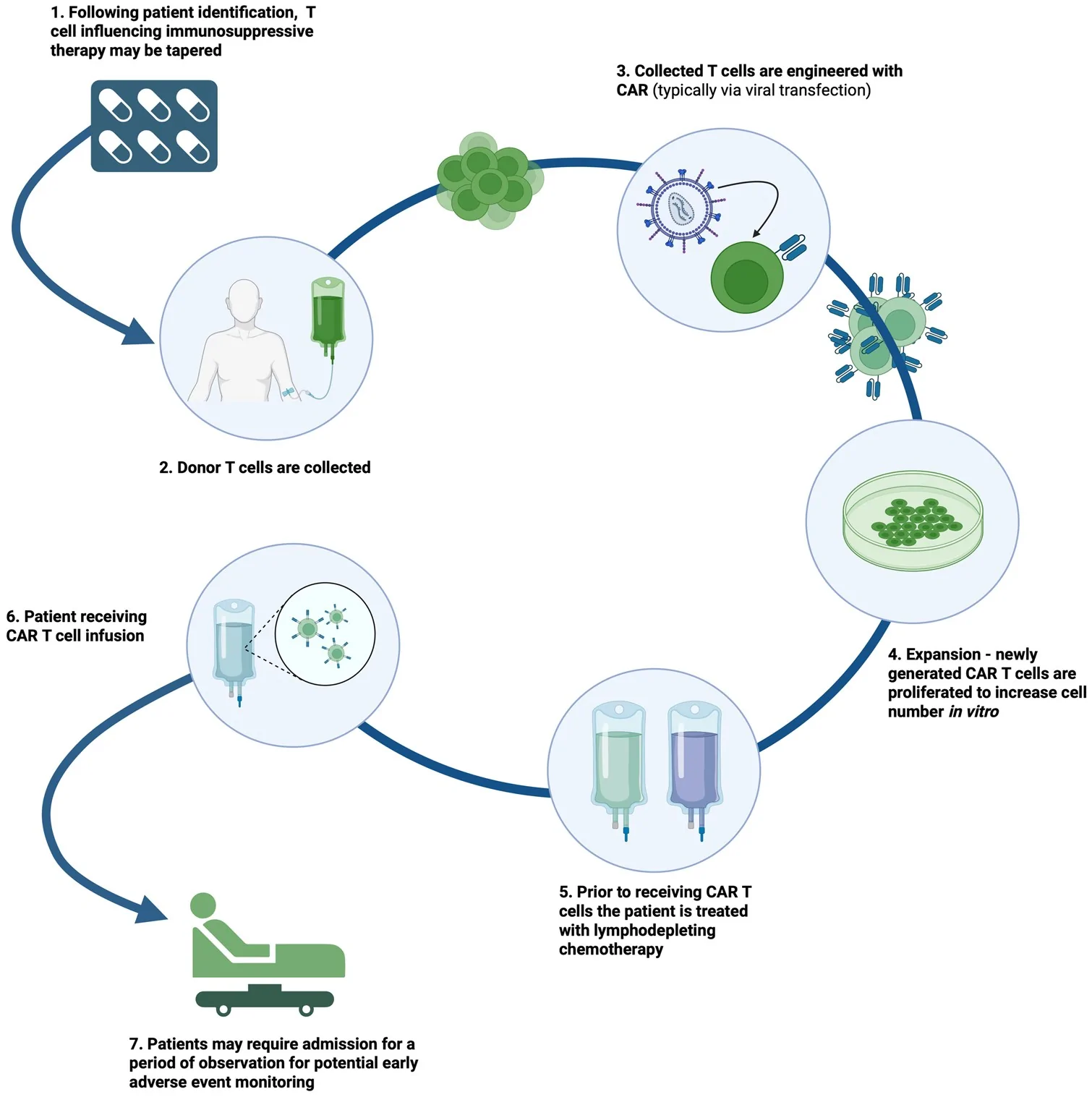
Manufacturing steps required to produce autologous CAR T cells for use in the treatment of SLE. Abbreviation: CAR: chimeric antigen receptor. Created in BioRender. Wincup, C. (2026)

Autologous CAR T cell therapy has emerged as a novel immunotherapeutic strategy for SLE based on its capacity to induce rapid, deep and sustained depletion of the B cell lineages via the binding of the CAR to either CD19 or BCMA. The CAR T cell infusion is usually preceded by lymphodepletion therapy to facilitate the expansion of CAR T cells in the body of the patient. Manufacturing of autologous CAR T cells takes several steps, which are summarized in Fig. 2. In SLE, CAR T cells have demonstrated rapid and profound deep B cell depletion accompanied by marked reductions in several autoantibody titres, normalization of complement levels and achievement of drug-free clinical remission [73] in the majority of patients. Importantly, these responses were sustained well beyond the period of detectable CAR T cell persistence, supporting the notion that transient CAR T cell activity can induce durable immunological reprogramming. Subsequent published experience has reinforced the feasibility and reproducibility of this approach across small cohorts, establishing proof-of-concept for CAR T cell therapy as a transformative modality in autoimmune disease [74–76]. Combined targeting of both CD19 and BCMA has also been recently investigated in an open label phase I study, in which the therapy was both effective and safe but was associated with episodes of hypogammaglobulinaemia [75]. Results of the first phase I/II trial with 24 autoimmune disease patients (including 10 SLE patients) treated with autologous CD19 CAR T cell therapy confirmed data on the safety and efficacy of CAR T cells from prior case series. Critical knowledge gaps currently remain, particularly regarding patient selection, timing of intervention and mechanistic predictors of durable response [77]. Current data are drawn from several small cohorts and early phase clinical trials. As the field continues to expand, further details will become available to allow assessment of long-term efficacy and safety of the therapy as more global data are collected.

Collectively, these early data shift therapeutic ambition in SLE from incremental disease control toward the possibility of long-term disease remission and potentially even cure. Immunologically, remission following CD19-CAR T cell therapy was associated with marked downregulation of type I IFN signalling, complement activation, chemokine networks and DNA damage response pathways. Compared with remission induced by rituximab or belimumab, CAR T-treated patients demonstrated deeper and broader suppression of IFN and chemokine signatures, alongside upregulation of lipid metabolic pathways consistent with immune quiescence. These findings suggest that CAR T therapy induces a qualitatively distinct immune reconstitution, characterized by more complete reconfiguration of pathogenic inflammatory circuits, which may underpin its durable efficacy [78].

Autologous CAR T cell therapy is currently the best-characterized cell-therapeutic approach in SLE, but faces practical barriers including time of manufacturing, costs and the need for specialized infrastructure. Allogeneic CAR T or CAR NK cell therapy aims to provide ‘off-the-shelf’ products [79, 80]. Notably, recent reports describe the induction of remission of SLE with allogeneic CD19-CAR T cells, though more data are need to judge the efficacy of this approach [81]. CAR NK cells are also being explored as a potentially safer approach, but efficacy data are needed to evaluate the potential of NK cells to induce long-lasting effects [82].

*In vivo* CAR T engineering is a transformative concept: instead of collecting and engineering cells *ex vivo*, lipid nanoparticles are infused that deliver CAR-encoding nucleic acid to endogenous T cells to express the CAR [83]. This approach could reduce the logistic barriers of cell manufacturing and improve the scalability of cell therapies for autoimmune diseases. A case series of five SLE patients treated with targeted lipid nanoparticles that trigger the generation of endogenous CD19-CAR T cells showed that this approach can trigger the formation of CAR T cells *in vivo* that were able to deplete B cells [84]. The kinetics of this *in vivo* CAR T cell approach is very different from *ex vivo* CAR T cells and it remains unclear at present how long the clinical effects of *in vivo* CAR T cell therapy might last.

### Safety considerations across modalities

The anticipated benefits of B cell-depleting therapies in SLE also require careful evaluation of potential risk of the therapy, which varies upon treatment modality. Real-world experience with rituximab has demonstrated that infusion-related reactions are common and frequently associated with the generation of anti-drug antibodies [85, 86]. In addition, late onset neutropenia has been observed in up to 20% of patients treated [87] and the risk of hypogammaglobulinemia accumulates with repeated cycles, carrying a risk of recurrent and serious infections [88, 89]. Similarly, obinutuzumab carries a risk of infusion-related reactions, although extended real-world data are still pending following licensing [61, 63].

Cellular therapies introduce a qualitatively distinct safety profile from monoclonal antibody therapies. The principle acute events are cytokine release syndrome (CRS) and immune effector cell associated neurotoxicity syndrome (ICANS). These have been graded in terms of severity by the American Society for Transplantation and Cellular Therapies [90]. In the haematological setting, CRS typically arises within 1–4 days post-CAR T infusion. Symptoms most commonly include fever, but more severe cases can result in haemodynamic compromise and hypoxia. ICANS can follow CRS but typically occurs later post-infusion, presenting with confusion, aphasia, tremor, seizure and reduced conscious levels [91]. Currently, the published literature for safety of CAR T cell therapy in autoimmune indications is reassuring but requires further data as more patients are treated. The CASTLE trial and preceding cases series have shown CRS has predominantly been low grade with ICANS being rarely seen [77]. Local immune effector cell-associated toxicity syndrome (LICATS) is a further recently described toxicity specific to the autoimmune setting. In an observational study of 39 patients with autoimmune disease (including 20 with SLE) treated with CD19-directed CAR T cells, transient organ-specific reactions occurred in 77%, arising at a median of 10 days post-infusion, confined to the period of B cell aplasia and, critically, restricted to organs already affected by the underlying disease, most frequently skin and kidney [92]. The large majority were mild and self-limiting, resolving without sequelae. LICATS thus appears mechanistically distinct from CRS and ICANS. Its recognition matters chiefly to avoid misattribution of these events to disease flare or intercurrent infection.

The medium-to-longer-term potential adverse effects of CAR T cell therapy are arguably more relevant to rheumatology. Lymphodepleting therapy may cause transient (and occasionally prolonged) cytopenias [93]. In addition, CD19-directed CAR T cell therapy results in B cell aplasia and a deeper level than monoclonal antibodies are able to achieve, potentially increasing the risk of infection [94]. Furthermore, BCMA-directed approaches additionally deplete antibody-secreting plasma cells and may produce pronounced hypogammaglobulinaemia [95]. Together, this heightened susceptibility to infection should be regularly screened for and managed proactively in regular follow-up care post-CAR T cell infusion. Prophylaxis principles drawn from haematology will require adapting for autoimmune indications as more data emerge but provide a helpful framework for current infection prevention strategies [96, 97].

### Considerations for inadequate response or relapse after cellular therapy

With positive data continuing to grow over the past 5 years, response data for CAR T cell therapy in autoimmune disease have accumulated rapidly, and inadequate response or relapse should be contextualized within that growing evidence base. SLE is sufficiently heterogeneous, both clinically and biologically, that uniform efficacy across patients is unrealistic. A sub-optimal response in any individual patient cannot be taken, in isolation, as failure of the modality, instead such cases warrant a structured diagnostic approach.

The first step is to confirm whether adequate B cell depletion was achieved, which requires the depth of peripheral B cell depletion, the kinetics of CAR T cell expansion and persistence, and, where feasible, the extent of tissue B cell depletion on biopsy to be assessed prospectively during the aplasia phase rather than at relapse, as B cells have reconstituted in nearly all reported cases by the time disease recurs [98, 99]. Together these readouts separate pharmacodynamic failure, in which insufficient B cell killing or premature CAR T cell exhaustion has occurred, from biological failure, in which disease persists despite effective depletion. Once pharmacodynamic effect is confirmed, attention should shift to the residual disease compartment. Persistent autoantibody production with high circulating plasmablast frequency implicates long-lived plasma cells, and a sustained type I IFN signature in the absence of major B cell-driven end point involvement points to the IFN axis. Ongoing tissue inflammation on repeat biopsy, particularly where lymphoid niches remain intact, signals compartments that are inaccessible to systemic therapy.

Second-line therapy should then be matched to the biology identified at that stage. Where long-lived plasma cell dominance is the most likely residual driver, BCMA-directed therapy via a T cell engager or CAR T cell is mechanistically rational [68, 70, 76]. However, the current clinical trial architecture excludes most patients with inadequate response or relapse after advanced cellular therapy for retreatment, which limits the evidence base on which any second-line algorithm can be built.

## Who should receive B cell-directed therapy? Toward precision stratification

The therapeutic targeting of B cells in SLE has expanded rapidly over the past decade, but the central clinical challenge has shifted from *whether* to target B cells to *who*, *what* and *how deeply* to target these cells. Not all active SLE patients may be equally B cell dependent, as in some patients B cell activation may be largely secondary to upstream inflammatory programmes (e.g. IFN and myeloid-driven networks). In this case, B cell depletion may only produce partial or transient benefit. Patient stratification should therefore address two linked questions. First, which pathogenic B cell compartment is dominant, including mature circulating B cells sustaining B–T collaboration, BAFF-dependent repopulating transitional/naïve populations that may drive autoreactivity after depletion, or long-lived plasma cells embedded within tissue niches. Second, what depth and modality of intervention is required to durably disrupt that compartment, ranging from functional B cell modulation (e.g. BAFF blockade), through conventional anti-CD20 monoclonal antibody depletion, to deeper lineage strategies such as TCE or CAR T cell therapies that extend into memory and precursor compartments. Addressing these questions will be essential to move beyond empirical depletion toward a biologically matched approach that maximizes efficacy while avoiding unnecessary intensity in patients whose disease is not primarily sustained by B cell-dependent circuits.

### Clinical phenotypes and biological plausibility

Until recently, B cell-depleting therapies have been preferentially deployed in patients with organ-threatening or treatment-refractory disease [49]. This has typically been in a setting in which conventional immunosuppression has failed to durably suppress disease activity, raising the likelihood that self-sustaining adaptive immune circuits (often B cell-dependent) are operative. However, in current practice this encompasses substantial biological heterogeneity. In some patients, ongoing disease activity may be driven by activated mature B cells and their interactions with T cells; in others, pathology may be maintained by plasma cell niches or tissue-resident immune organization that is relatively resistant to conventional B cell-depletion strategies. Precision deployment must therefore consider biological mechanisms and not clinical phenotypes alone. There is additional growing evidence to support the use of B cell-targeting therapies earlier in the disease process and in those with more moderate manifestations of the disease, thus suggesting a realignment of these therapies in the disease course should be considered [51, 100, 101].

### Stratification for sequential therapy: lessons from rituximab–belimumab combination

One of the clearest opportunities for biologically informed stratification arises in the post-depletion setting. Anti-CD20 therapy induces a marked rise in circulating BAFF concentrations, creating conditions that favour survival of autoreactive B cells during reconstitution. The rationale for belimumab following rituximab is therefore mechanistic, to reshape the repopulating B cell repertoire and reduce relapse risk [9].

The BEAT-LUPUS trial provided a proof-of-concept that this strategy can improve clinical outcomes. Whilst the primary outcome of the study was change in anti-dsDNA titres, key secondary endpoints also demonstrated reduced flares and no abnormal safety signals with belimumab compared with placebo after rituximab [102]. Importantly, mechanistic analyses from BEAT-LUPUS have gone further, identifying candidate biomarkers that may predict benefit from belimumab in the context of B cell reconstitution. Baseline IgA2 anti-dsDNA antibodies, increased IgA2 plasmablasts and expanded T follicular helper cells are promising biomarkers predicting clinical response to belimumab following rituximab, with these markers selectively declining in responders receiving belimumab [103]. Further mechanistic data are provided by analysis from a phase II proof-of-concept study of combined rituximab and belimumab, in which dual targeting achieved profound and sustained B cell depletion, with a median 97% reduction in CD19⁺ B cells at 24 weeks and persistent suppression (84%) to 2 years. Importantly, the addition of belimumab appeared to prevent full post-rituximab B cell repopulation, particularly limiting early re-emergence of double-negative B cells, which repopulated more rapidly in non-responders [104].

### Emerging considerations for T cell engagers and CAR-based therapies

Formal predictor datasets for TCEs and CAR-based cellular therapies in SLE remain limited due to low numbers of patients treated to date; however, they are growing, with early clinical trial data recently emerging [77, 105]. Early SLE CAR T cell therapy experience suggests that efficacy extends to rituximab failures [73, 75]. Critically, these considerations must be interpreted in the context of tissue-resident B cell populations, as multiple studies have demonstrated that pathogenic B cells persist within secondary lymphoid organs and inflamed tissues, including lymph nodes and kidney, where accessibility to conventional antibodies is incomplete. However, deep B cell depletion within lymphatic tissue has been demonstrated following cellular therapy in SLE [98, 99].

The current lack of systematic data linking lymphoid tissue depletion, plasma-cell targeting, and cellular therapy outcomes represents a major knowledge gap, underscoring the need for integrated biomarker strategies that extend beyond peripheral blood to guide the rational deployment of next-generation cellular therapies in SLE [106].

### Beyond proof-of-concept: priorities for trial design, patient selection and access to therapies

The early phase cell therapy pipeline in SLE has expanded substantially over recent years, with multiple academic and commercial studies either underway or in development. However, as data emerge there is a risk of slow accrual of generalizable evidence. Head-to-head randomized trials directly comparing monoclonal antibodies, TCEs and CAR T cell therapy are unlikely to be feasible at the sample sizes required, and further still may not be acceptable for participants approached for such studies.

Furthermore, greater emphasis is required for equity and access to care. There is pressure on apheresis infrastructure, inpatient bed capacity limitations and the requirement for hospitalization post-CAR T cell therapy. SLE often carries greatest risk of both morbidity and mortality in patients of African, Asian and Hispanic heritage, who frequently experience more severe organ manifestations and poorer outcomes [107, 108]. In their current form, cellular therapies will be challenging to deliver for many patients in low- and middle-income healthcare systems. Furthermore, manufacturing, lymphodepleting conditioning, intensive monitoring and infrastructure required to manage CRS and ICANS may initially only be concentrated within a small number of highly specialized centres. It is therefore essential that equity of access is placed as a high priority for future strategies. Deliberate action will be required to ensure that the most highly effective modalities of therapy reach the patients of greatest need. Scalability of cellular options with be of high priority. However, the landscape is likely to change significantly in the coming years, with biosimilar access to biologics, ‘off the shelf’ allogeneic CAR and *in vivo* CAR engineering all likely to improve access to more widespread use of many modalities of B cell depletion.

## When to intervene? Timing, disease course and immune plasticity

The timing of B cell-directed therapy is a critical, yet often underappreciated, determinant of clinical outcome in SLE. Disease duration, cumulative organ damage and the degree of established immune pathways profoundly influence both therapeutic and immunological responsiveness that can be achieved.

### Early disease and immune plasticity: a window for biologic intervention

Early, treatment-naïve SLE is not immunologically quiescent, with multiple studies at diagnosis showing that pathogenic B cell pathways are already active, but their relative dominance varies between patients [109–111]. In a cohort of treatment-naïve childhood-onset SLE, high-dimensional profiling demonstrated expansion of EF B cell subsets (including plasmablast-associated states) and peripheral T helper cells, alongside heightened inflammatory activation at disease onset, supporting early establishment of EF circuits [112]. Mechanistically, EF ‘pre-plasma cell’ trajectories are consistent with the TLR7-driven DN2–CD11c⁺T-bet⁺ B cell axis [113]. Treatment-naïve, new-onset cohorts also show early reshaping of the compartment with reduced memory pools and increased naïve/double-negative populations compared with controls [114]. Importantly, ‘early SLE’ is noted to be biologically heterogeneous at baseline with treatment-naïve patients segregate into distinct lymphocyte subset clusters associated with different clinical phenotypes, arguing that the key issue is not ‘plasticity’ *per se* but which B cell activation pattern is dominant in a given patient [115]. This biological context provides a strong rationale for earlier use of effective B cell-directed therapies, particularly monoclonal antibodies that modulate B cell survival or deplete mature compartments.

Similarly, in LN early introduction of biologic therapy on top of standard immunosuppression appears to improve outcomes [116, 117]. These data support a front-loaded strategy, in which early, effective B cell targeting reduces inflammatory burden, limits organ damage and potentially prevents maturation of resistant immune compartments.

### Delayed intervention and the biology of established disease

In contrast, long-standing SLE is marked by progressive immunological consolidation. Over time, autoreactive memory B cells, long-lived plasma cells and tissue-resident immune structures become increasingly stable. Tertiary lymphoid structures may form within target organs, providing local niches for antigen presentation and differentiation that are relatively insulated from systemic therapies [42, 43]. In parallel, cumulative exposure to immunosuppressive agents alters immune cell fitness, particularly within the T cell compartment.

These changes have important implications for timing; for example, in established disease, conventional biologics may still suppress inflammation and reduce flare frequency, but their capacity to fundamentally reprogramme disease biology is likely diminished. Depletion of circulating B cells may not adequately disrupt tissue-resident pathogenic circuits, and BAFF modulation may have limited impact once autoreactivity is sustained by plasma cell-dominant processes. This helps explain why some patients experience transient benefit from B cell-directed therapies but relapse as pathogenic compartments reassert dominance.

### Timing considerations for cellular therapies

Cellular therapies offer the advantage of deep lineage targeting. In the first case series, SLE patients had highly treatment-refractory disease with patients cycling through virtually all drugs before being exposed to CAR T cells. In the recent CASTLE trial [77] and in the first recommendation from the European Society for Bone Marrow Transplant (EBMT), earlier exposure to CAR T cell therapy (after failure of only two treatments) has been suggested as an approach to prevent organ damage as soon as possible. The recently published German SLE guidelines included cell therapy approaches for the first time [118]. Too-late deployment of CAR T cell therapy may introduce other limitations, particularly organ damage and reduced T cell fitness [119, 120]. In patients with long-standing SLE, those exposed to prolonged high-dose glucocorticoids, cytotoxic agents or repeated biologic therapies, T cells may exhibit features of exhaustion, senescence or impaired metabolic capacity [121–123]. This may compromise cellular therapy efficacy and increase variability in expansion, persistence and clinical response.

With CAR T cell treatment, lymphodepleting therapy (required for most CAR T therapies) adds an additional layer of complexity in patients with time-limited immunosuppression and risk of infection. On the other hand, the withdrawal of glucocorticoids and conventional immunosuppressants may also lower the infection in the long run. Strategies that target plasma cells (i.e. BCMA-targeted TCSs and CAR T cells) may result in sustained hypogammaglobulinaemia, impaired vaccination responses and increased susceptibility to infection. The clinical challenge is therefore to balance early procedural and immunological risks against the potential long-term benefit of immune reconstitution and reduced cumulative immunosuppressive exposure, with careful longitudinal monitoring of immune competence.

Finally, deeply established plasma cell niches and tissue-resident immune architecture may be less amenable to eradication in late disease, even with potent cellular approaches. While early case series demonstrate remarkable responses in selected refractory patients, it remains unclear whether similar outcomes can be achieved broadly once disease biology is fully consolidated, particularly given that early trial experience has been in patients with comparatively short disease duration. Further data are required before the optimal position for cellular therapies in SLE is known.

As B cell-directed therapies continue to diversify, prospective studies explicitly incorporating disease duration, immune fitness and tissue biology will be essential to define not only *who* should receive which therapy, but *when* that therapy is most likely to succeed.

## Conclusion

B cell-directed therapy has emerged as a central pillar of therapeutic innovation in SLE evolving from monoclonal antibodies to T cell-redirecting agents and cellular immunotherapies; however, biomarkers remain to be established that can better predict response. Furthermore, greater emphasis will need to be placed on the timing of therapeutic intervention. Early disease may offer a window of immune plasticity in which biologic intervention can modify trajectory, whereas established disease is marked by immunological consolidation and tissue residency that need other therapeutic approaches. Together, these insights expose the limitations of phenotype-driven treatment and highlight the need for biological stratification.

Taken together, the evidence supports a precision-medicine framework in which B cell-directed therapies are selected according to *the right patient*, with *the right drug* and at *the right time*. Embedding mechanistic biomarkers into clinical trials and practice will be essential to realise the full disease-modifying potential of B cell targeting in SLE.

## Data Availability

No new data were generated or analysed in support of this work.
